# Dulcamara

**Published:** 1887-08

**Authors:** P. P. Wells

**Affiliations:** Brooklyn, N. Y.; Bureau Materia Medica, I. H. A.


### DULCAMARA.

P. P. WELLS, M. D., BROOKLYN, N. Y.

(Bureau Materia Medica, I. H. A.)

I first saw the Dulcamara in fruit in 1846. It was growing near and spreading over a stone wall which surrounded land now covered by city residences, having since become a part of Brooklyn. I broke and carried away some branches bearing the berries, and while walking toward my home, some three-fourths of a mile distant, I chewed one of the berries till its peculiar bitter-sweet taste became a little unpleasant, when it was thrown out, and besides this taste nothing more was looked for. But in about five minutes I became aware of another impression, a slight nausea and general uneasiness, which was almost immediately followed by violent, spasmodic, suffocative coughing. It almost took my breath away. These paroxysms were repeated every three or four hours for two weeks, when they were arrested by other medicine. They were accompanied by the peculiar resonant inspiration or whooping characteristic of the cough called by this name. The paroxysms were excited by any attempt at loud speaking or even the slightest movement toward laughing.

They followed almost immediately after eating, with retching, and too often this resulted in the loss of what had been just taken with good appetite. The paroxysms sometimes attacked me while eating and spoiled the disposition to continue this useful employment for that time. These paroxysms were only experienced in the daytime. There was no coughing at night. They were always accompanied with violent strangulation, and respiration was only regained by the characteristic whooping inspiration we all know so well. They were always accompanied by violent retching and frequently by vomiting, and, if near the time of eating, by loss of food.

Was this experience a proving of *Dulcamara?* Before an answer to this question it is right to consider some circumstances which were present when this experience was passed through.

First—There was an epidemic of whooping cough prevailing in Brooklyn when the berry was chewed. Second—There were two examples of it in my family, of three weeks' standing at the time, so I had been in the atmosphere for this time. Third—Some people repeat the paroxysms of the cough on exposure to its infection, even though they have previously gone through the usual course of the disease. Some are so susceptible to the action of this specific noxia as to take on the peculiar habits and phenomena of the disease whenever they are exposed to its presence. I have had patients who have so suffered many times. Fourth—I had whooping cough in early life. I had been many times exposed to its course in my own family, as in this case, and in many others, both before and after this singular occurrence, with no apparent effect resulting from this.

Then the suddenness of the first attack after eating the berry. It was in less than five minutes, and in force in full power. No subsequent one was more violent than was the first, and this is not the mode of attack of whooping cough caused by its specific noxia. Its initiatory paroxysms are comparatively light, but as the disease progresses these increase in violence, as was not the case with those which followed so soon after chewing the berry.

But the question may be asked, Was there not in my organism a susceptibility to the action of the *Dulcamara* induced by my protracted exposure to the epidemic atmosphere and the concentration of the poison in my own home, so that by reason of these facts I was, when I ate the berry, on the verge of the attack, which was only set in motion by the presence of the *Dulcamara?* If the affirmative of this suggestion be accepted, then the train of phenomena which followed constitutes an actual proving of the drug and establishes its theraputic relationship to whooping cough. If this view be accepted it proves a similarity of the action of the drug to the specific action of the dynamis which causes the phenomena of the disease, and therefore commends it to our notice as one of the remedies for this sometimes troublesome plague.

The recital of this case is not given to the Association as a perfected proving of the drug, but as a suggestion of its relationship to a painful malady as its possible curative, of sufficient import to call for a more complete proving of the drug with a view to ascertaining whether this hitherto unsuspected relationship be a truth which may be added to our materia medica record for its enrichment and greater power as healers over an important and sometimes fatal malady.

There are some other considerations which strengthen the suggestion of this experience being a true proving of Dulcamara. It did not take the form of the epidemic then prevailing, but differed from this in material points. There was in this case *no cough at night.* The cases of the epidemic were worse at night. This case was strongly marked by the cough excited by *loud speech* and the least *movement toward laughter.* The remedy for that epidemic was Ipecacuanha, which has no such symptoms; neither had the epidemic. If this series of paroxysms of spasmodic suffocating cough were the result of the epidemic noxia then active in the community, they could hardly have differed from those of the epidemic in so important characteristic symptoms as were the case in this wholly unlooked-for and troublesome experience.

And, further, if this excellent imitation of whooping cough was the effect of the *Dulcamara* it is of peculiar value by reason of the fact that its paroxysms were only experienced *in the daytime,* while the remedies most frequently called for in the treatment of epidemics of this cough are characterized by *worse at night and when lying down.* In this case there was no cough when lying down.

